# Allatotropin Modulates Myostimulatory and Cardioacceleratory Activities in *Rhodnius prolixus* (Stal).

**DOI:** 10.1371/journal.pone.0124131

**Published:** 2015-04-21

**Authors:** María José Villalobos-Sambucaro, Alicia Nieves Lorenzo-Figueiras, Fernando Luis Riccillo, Luis Anibal Diambra, Fernando Gabriel Noriega, Jorge Rafael Ronderos

**Affiliations:** 1 Cátedra Histología y Embriología Animal (FCNyM-UNLP), La Plata, Argentina; 2 Centro Regional de Estudios Genómicos (CREG-UNLP), La Plata, Argentina; 3 Department of Biological Sciences, Florida International University, Miami, Florida, United States of America; 4 Departamento de Biodiversidad y Biología Experimental (IBBEA, FCEyN-UBA), Buenos Aires, Argentina; Universidade Federal do Rio de Janeiro, BRAZIL

## Abstract

Haematophagous insects can ingest large quantities of blood in a single meal and eliminate high volumes of urine in the next few hours. This rise in diuresis is possible because the excretory activity of the Malpighian tubules is facilitated by an increase in haemolymph circulation as a result of intensification of aorta contractions combined with an increase of the anterior midgut peristaltic waves. It has been previously described that haemolymph circulation during post-prandial diuresis is stimulated by the synergistic activity of allatotropin (AT) and serotonin in the kissing bug *Triatoma infestans*; resulting in an increase in aorta contractions. In the same species, AT stimulates anterior midgut and rectum muscle contractions to mix urine and feces and facilitate the voiding of the rectum. Furthermore, levels of AT in midgut and Malpighian tubules increased in the afternoon when insects are getting ready for nocturnal feeding. In the present study we describe the synergistic effect of AT and serotonin increasing the frequency of contractions of the aorta in *Rhodnius prolixus*. The basal frequency of contractions of the aorta in the afternoon is higher that the observed during the morning, suggesting the existence of a daily rhythmic activity. The AT receptor is expressed in the rectum, midgut and dorsal vessel, three critical organs involved in post-prandial diuresis. All together these findings provide evidence that AT plays a role as a myoregulatory and cardioacceleratory peptide in *R*. *prolixus*.

## Introduction

Juvenile individuals of the kissing bug *Rhodnius prolixus* (Stal) (Hemiptera: Reduviidae) can ingest a volume of blood up to 12.5 times its unfed weight in a single meal [1]. Consequently large quantities of salts and water must be quickly eliminated in the urine in order to decrease the weight and restore water and mineral balance. Large volumes of urine are produced during the first few hours after feeding [2–6]. Malpighian tubules (MTs) respond by increasing their rate of secretion to produce hypo-osmotic urine and re-establish the osmotic balance [2, 7]. This physiological process is controlled by diuretic and antidiuretic hormones; being serotonin an important regulator of MTs activity [7–9]. Water and ion homeostasis also depends on the ability of the dorsal vessel (DV), composed by the heart and the aorta, to pump haemolymph in a posterior-anterior direction from the abdominal segment 6^th^ along the abdomen to the thorax and head [10]. Since the pioneering studies of Gerould [11] it is known that in several insect species the heart beat alternates between anterograde and retrograde directions. In the mosquito *Anopheles gambiae*, this reversal pattern of contractions is restricted to the adults [12]. It was later confirmed that this alternating pattern of contractions is also present in other taxa including Orthoptera and Hemiptera [13]. On the contrary, in *R*. *prolixus* the DV normally pumps haemolymph only in a postero-anterior direction. Diuresis also depends on the ability of the anterior midgut (crop) to move haemolymph in an antero-posterior direction [2]. In fact immediately after the ingestion of blood, the number of peristaltic waves of the crop increases, facilitating haemolymph recirculation and diuresis [2].

In several insect species serotonin is also involved in the regulation of visceral and cardiac muscle contractions. Serotonin increases the heart beat frequency of *Drosophila melanogaster* larvae [14]. In *Agrius convolvuli* larvae (Lepidoptera: Sphingidae), serotonin increases the rate of anterograde contractions, and reverses to anterograde direction when applied during the posterograde phase [15]. Serotonin regulates several physiological processes in *R*. *prolixus*. In addition to the role as a diuretic factor, serotonin controls other processes during feeding, including salivation and plasticization of the cuticle [9], serotonin increases the heart rate of contractions at a concentration of 10^-8^ M [16].

Allatotropin (AT), a neuropeptide isolated on the basis of its activity stimulating juvenile hormone synthesis in the lepidoteran *Manduca sexta* [17], has also proved to be multifunctional, acting in different insect species as myoregulator and cardioaccelerator [18–22]. In *R*. *prolixus*, AT has no effect modulating heart beat frequency or contractions of the digestive tract under basal conditions [23]. In the related species *Triatoma infestans* AT increases the frequency of contractions of the DV, crop and hindgut [24, 25]. In unfed adult males of *T*. *infestans*, AT has no myoregulatory effect by itself, but synergizes the stimulatory effect of serotonin on the frequency of the DV contractions [25]. Previous studies described that AT released by the MTs controls rectum muscle activity, stimulating peristaltic waves that mix and helps voiding urine and feces [24, 26]. Treatment of 4^th^-instar larvae with AT-antiserum decreased urine elimination [24]. In *T*. *infestans*, AT is present in the nervous system, midgut epithelium and MTs [25–27]; and the production of the peptide in these tissues undergoes daily rhythmic activities, with highest levels on the afternoon as insects prepare to blood-fed [28].

In the present study, we provide evidence that in *R*. *prolixus* AT increases the frequency of contractions of the aorta only when insects have been previously treated with serotonin; suggesting a synergistic relationship between these two hormones. The expression of the AT receptor in the target organs (i.e. hindgut/rectum, DV and midgut); and the blockade of urine voiding with AT antiserum, suggest that as in *T*. *infestans*, this peptide is involved in physiological processes associated with post-prandial diuresis, facilitating haemolymph recirculation and deposition of urine and feces. 

## Material and Methods

### 2.1 Insects

Adult males and 4^th^-instar larvae of *R*. *prolixus* were obtained from a colony maintained at 28 ± 2°C, 45% relative humidity, and 12:12 hour light-dark period. All the experiments were performed *in vivo*. Adult males were immediately isolated after molting and starved during 14 to 21 days before experiments were performed. 4^th^-instar larvae were immediately isolated after molt (3^rd^ to 4^th^ instar) and also starved during 14 to 21 days. All the experiments were performed with experimental groups conformed by 6 to 14 insects. Insects were fed on phosphate buffer saline in an artificial feeder and immediately sacrificed after the experiments.

### 2.2 Myoregulatory bioassays

The effect of AT on the contractions of the aorta and the anterior midgut was analyzed *in vivo*. To perform these experiments, the wings of the insects were removed to expose the dorsal cuticle of the abdomen. Due to the transparent nature of the cuticle, the contractions of the aorta and the peristaltic waves of the anterior midgut were clearly recorded [25] (S1 Movie). We tested the effect of *Aedes aegypti* AT (APFRNSEMMTARGF) (Biopeptide, San Diego, CA) [29] which shares a 58.3% identity and 83.3% similarity with the corresponding *R*. *prolixus* peptide. The concentrations tested were 10^-9^ and 10^-6^ M. Peptides were diluted in 3 μl of *R*. *prolixus* saline [4]. Controls received only saline. AT was administered through an incision of the conexive tissue in the first abdominal segment. To minimize the effect of the stress caused by handling, insects were rested for 30 minutes before the administration of the first treatment. The contractions of the aorta and peristaltic waves of the anterior midgut were observed through the dorsal cuticle (segments IV and V of the abdomen) under a dissection microscope. The number of contractions in a 3-min period was recorded at 5, 15 and 30 minutes after treatments [24, 25]. To evaluate the effect on the peristaltic waves of the crop, only those contractions that produce a complete anterior-posterior wave through the abdomen were recorded. Local contractions (usually observed at the level of the segments II and III of the abdomen) were not recorded. All data were collected by the same operator. Results are expressed as number of contractions or peristaltic waves per minute. For experiments involving fed insects, bugs were allowed to feed for 15 minutes, and only those insects fed *ad libitum* were selected.

### 2.3 Effect of feeding AT antiserum on diuresis

Insects were fed using an artificial feeder developed by Nuñez and Lazzari [30]. Groups of 4^th^-instar larvae of *R*. *prolixus* were fed: 1) buffer phosphate saline (PBS) (controls), 2) AT-antiserum whose specificity was previously confirmed [24, 26–27, 29] diluted in a PBS solution (1/100) [24], and 3) A solution containing AT-antiserum that was preadsorbed overnight at 4°C with synthetic AT (200 nmol) [24]. All insects were fed during 15 min, and only those insects fed *ad libitum* were selected. Each fed insect was individually placed in microtubes. The volume of urine was measured at 15, 30, 45, 60, 90, 120, 180, 240 min, and 24 h (1440 min). To calculate the quantity of urine produced, the weight of each microtube was recorded before the insect was caged in it. After each period (i.e. 0–15 mins; 15–30; etc.) the insect was removed from the tube and the weight of the tube was recorded. To evaluate the quantity of urine released by the insect, the final weight of the microtube was compared to the original weight. After that, each insect was placed in a new microcentrifuge tube previously weighed to evaluate changes in the next period. In addition, the frequencies of aorta contractions and crop peristaltic waves were recorded at 15, 30, 45, 60, 90, 120 and 240 min on male insects that were fed either PBS or a PBS-AT-antiserum solution.

### 2.4 Identification and characterization of the AT receptor

The sequence of the *Manduca sexta* AT receptor (ADX66344.1) was used to search the *R*. *prolixus* genome (https://www.vectorbase.org) using the TBLASTN algorithm and the BLOSUM62 matrix. The structure of the *RpATr* gene was predicted using the Augustus software (http://augustus.gobics.de).

Allatotropin receptor sequences were obtained from databases and used for the alignments. We aligned the allatotropin receptor sequences using Clustal W (http://www.ebi.ac.uk/Tools/msa/clustalw2/) and JalView 2.7 [31]. The accession numbers of the AT receptors utilized are: *Manduca sexta* (ADX66344.1), *Danaus plexippus* (EHJ74388.1), *Bombyx mori* (NP_001127714.1), *Aedes aegypti* (AEN03789.1), *Tribolium castaneum* (XP_973738.2), *Bombus terrestris* (XP_003402490.1), *Bombus impatiens* (XP_003486747.1), *Apis florea* (XP_003690070.1), *Megachile rotundata* (XP_003708421.1), *Harpegnathos saltator* (EFN76143.1), *Nasonia vitripennis* (XP_001604582.2), *Schistocerca gregaria* (AEX08666.1). The seven transmembrane domains of the AT receptors were determined using the online software *InterProScan* [32].

AT receptor mRNA tissue expression was studied using RNA extracted from whole 4^th^ instar larvae and adult *R*. *prolixus* Malpighian tubules, DV, hindgut/rectum, ovaries, and midgut. RNA was isolated using RNAeasy kit for RNA isolation according to the specifications of the manufacturers (Qiagen). The RNA was first treated with RNAse-free DNAse Set (Qiagen) to prevent amplification of genomic DNA. First strand cDNA was synthesized using Revert Aid First Strand cDNA Synthesis Kit (Fermentas, USA) and used as template in a PCR reaction with the following two set of primers:

1. Primer Forward 5´—ATGTCCGATGAAGACTATCTG—3´ and primer Reverse 5´—TGTAGATAAGAGGATTAGTGGC—3´.
2. Primer Forward 5´—ATGTCCGATGAAGACTATCTG—3´ and primer Reverse 5´—GTAGAGCACTAATTTGCAGAG—3´

The sequence of the PCR products was confirmed using cDNA sequencing (Unidad de Genómica—Instituto de Biotecnología—CICVyA—CNIA—INTA, Argentina).

### 2.5 Statistical analysis

Significant differences were evaluated by multifactorial Analysis of Variance (ANOVA) considering two factors acting on the evaluated variables (i.e. treatment and time of frequency recording). Single post-hoc comparisons were tested by the Tukey test. Only differences equal or less than 0.05 were considered significant. Data are expressed as means ± standard error.

## Results

### 3.1 Allatotropin does not increase the frequency of aorta contractions under basal conditions

**Fig 1:**
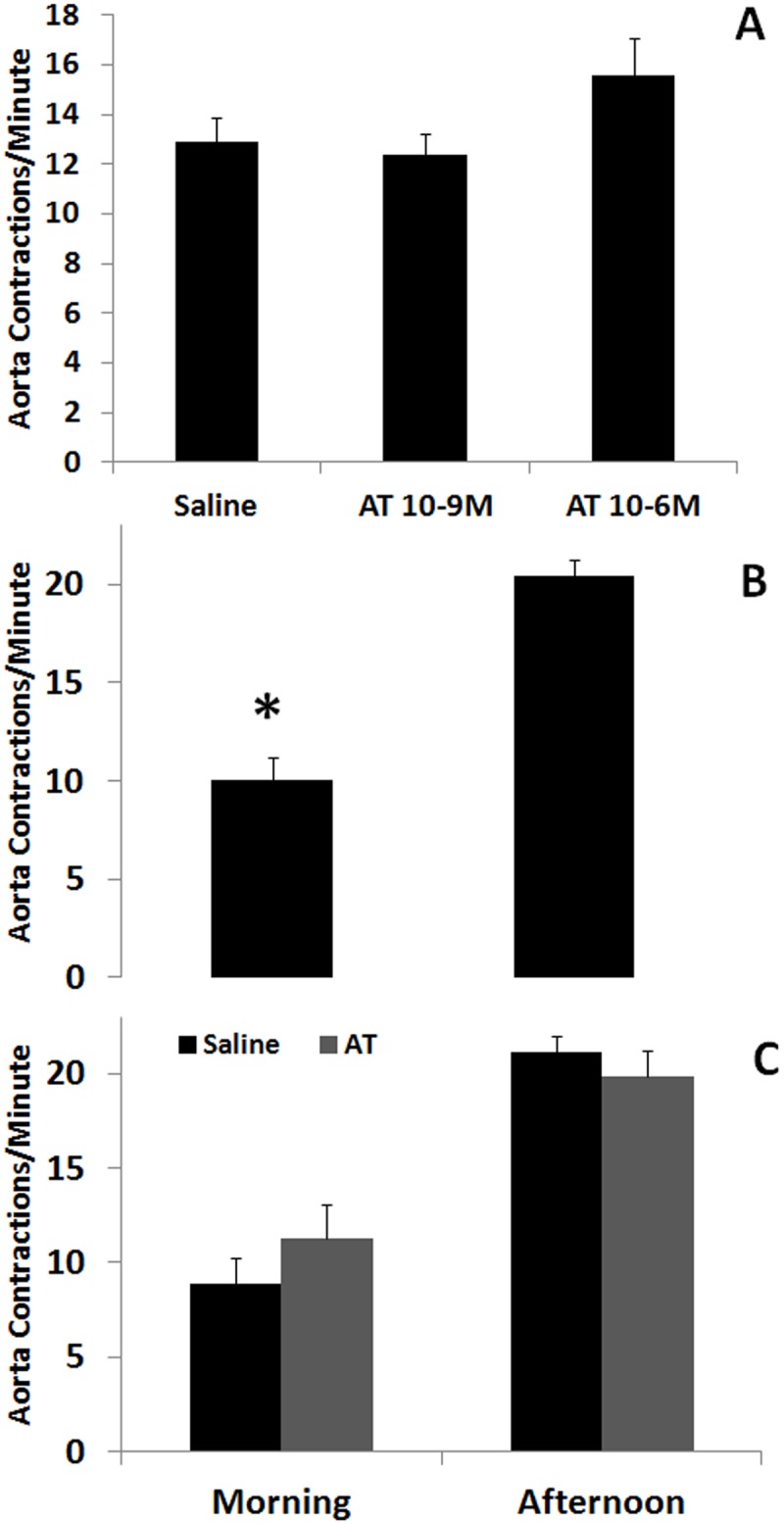
Daily rhythmic contraction of the aorta and AT effect on the basal contractions. **A)** Allatotropin did not show myoregulatory effect on the basal frequency of contractions of the aorta at doses of 10^-9^ and 10^-6^ M. **B)** Basal frequency of contractions of the aorta of unfed adult males measured during the morning and afternoon. **C)** AT (10^-6^ M) does not show myoregulatory activity on the basal frequency of contractions of the aorta when assayed during the morning or the afternoon. Each bar represents Mean ± standard error (n = 6 for each treatment). Asterisks represent statistically significant differences between frequencies.

Treatment with two different concentrations of AT (10^-9^ and 10^-6^ M) did not significantly increase the basal frequency (Fig 1A).

### 3.2 Daily rhythmic contraction of the aorta

The basal frequency of aorta contractions displayed daily rhythmic activity; with maximum frequencies in the afternoon (Fig 1B). To analyze variations in the activity of AT associated to daily rhythms, we tested the effect of 10^-6^ M AT in the morning (low rate) and in the afternoon (high rate). AT did not show effect neither in the morning nor in the afternoon (Fig 1C).

### 3.3 Allatotropin and serotonin synergistically increased aorta activity

**Fig 2:**
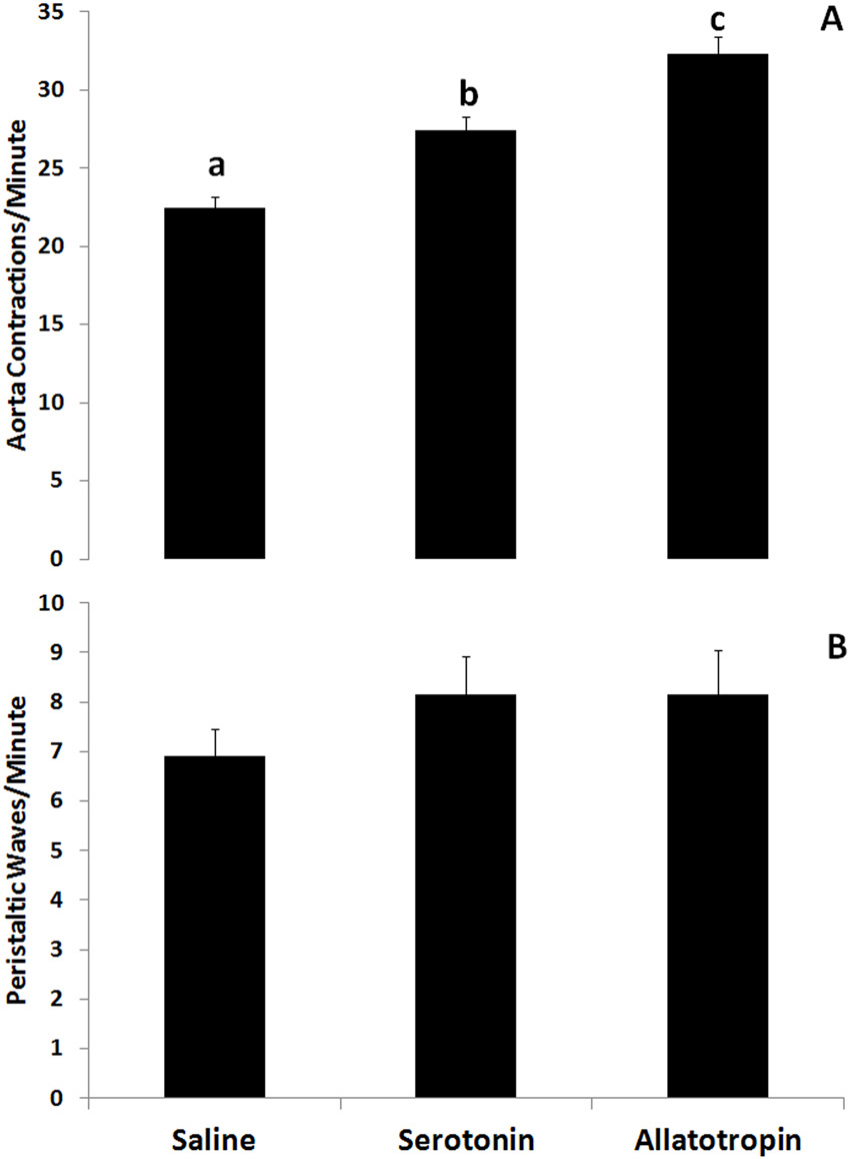
Synergistic activity of AT on the increase of the frequency of contractions of the aorta induced by serotonin. **A)** AT treatment (10^-9^ M) induces a further increase on the frequency of contractions of the aorta after the increment produced by serotonin (10^-9^ M). **B)** The rate of the peristaltic waves of the anterior midgut are not significantly modified by serotonin or AT treatments. Each bar represents Mean ± standard error. Different letters represent statistically significant differences between treatments (n = 6 for each treatment).

To test the synergistic effect of AT and serotonin on aorta contractions, insects were treated first with serotonin (10^-9^ M), and subsequently injected with AT (10^-9^ and 10^-6^ M). Serotonin increased the frequency of aorta contractions, and injection of AT 10^-9^ M resulted in an additional increment on the frequency of contractions (Fig 2A). The treatment with the highest dose previously tested (10^-6^ M) caused a maximum and sustained contraction of the DV (data not shown). In the same group of insects the rate of peristaltic waves of the crop was evaluated, but no significant differences were observed (Fig 2B).

### 3.4 Ingestion of AT antiserum decreased urine elimination, aorta frequency of contractions and rate of peristaltic waves of the crop

**Fig 3:**
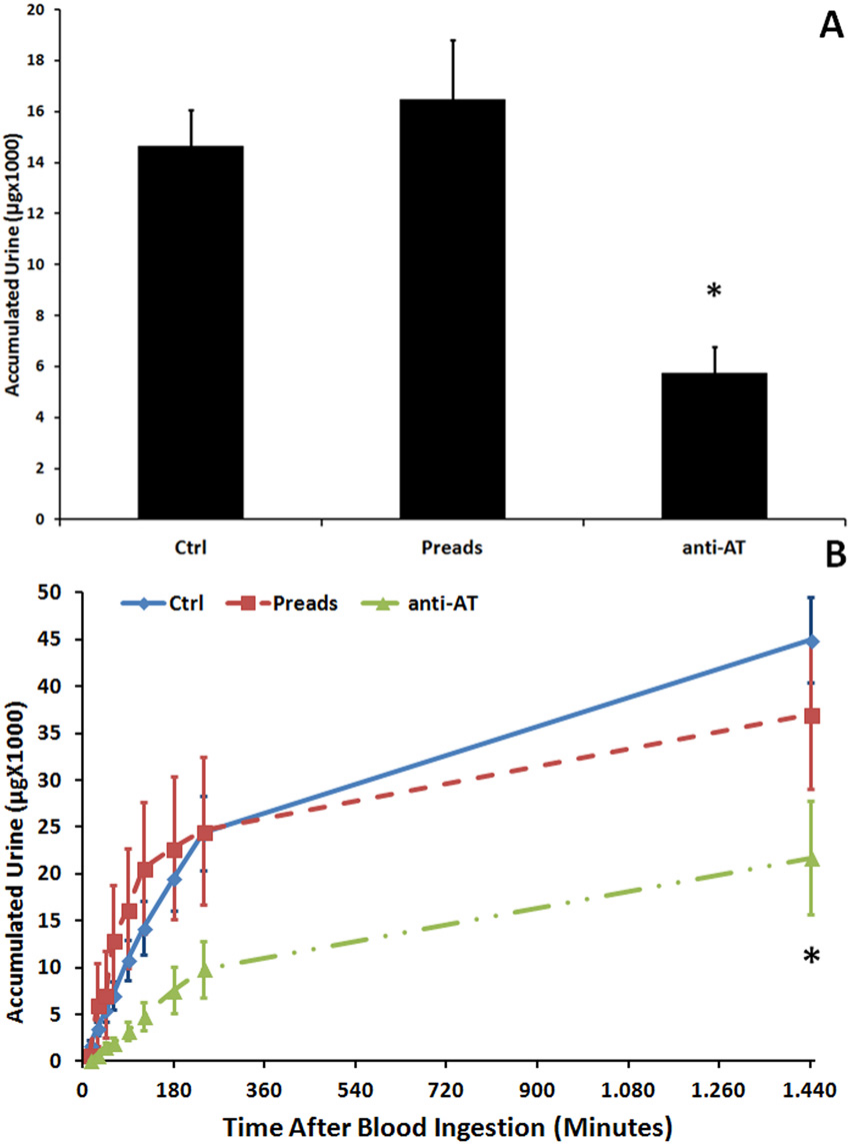
Allatotropin antiserum supplied with the meal decreases urine elimination, aorta frequency of contractions and rate of peristaltic waves of the crop. **A)** Effect of AT-antiserum on the urine released during the first 24 h (1440 min) after a blood meal. **B)** Effect of anti-AT supplied with the meal on the accumulated urine released by 4^th^-instar larvae. Each data represents Mean ± standard error. Asterisks represent statistically significant differences between treatments.

**Fig 4:**
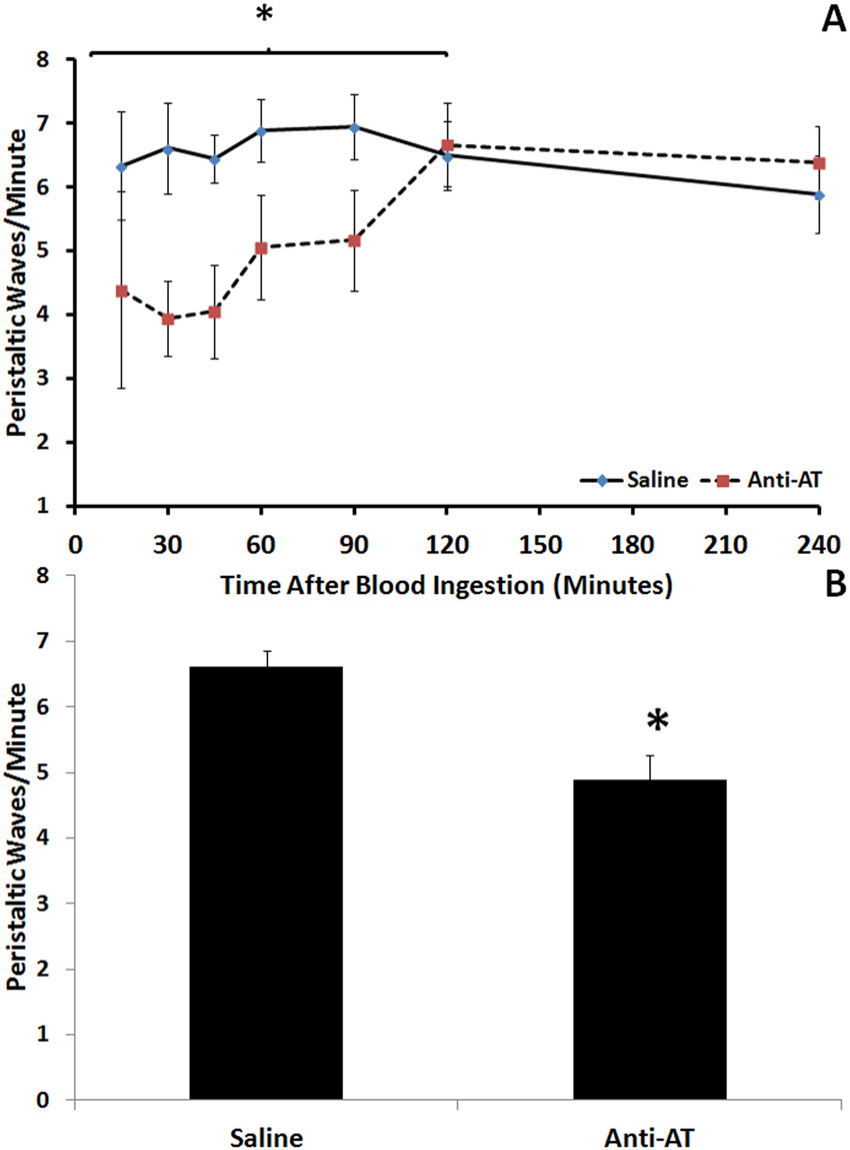
Inhibition of the peristaltic wave rate of the crop induced by the administration of the AT-antiserum with the meal. **A)** Peristaltic wave rate in each time recorded along 240 mins after meal. **B)** Comparison of the peristaltic wave rate of the crop along the first 120 mins after a meal. Each point represents Mean ± standard error. Asterisks in each graph represent statistically significant differences between treatments.

**Fig 5:**
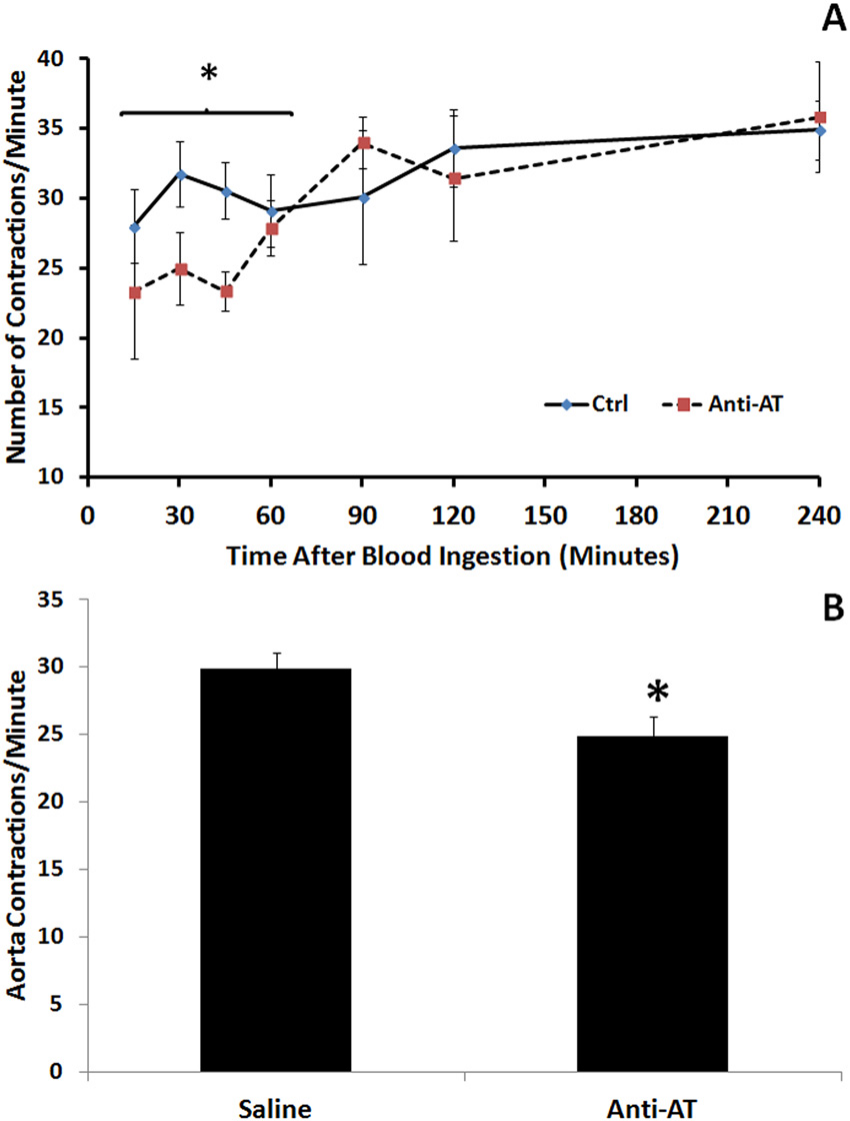
Decrease of the frequency of contractions of the aorta after the administration of the AT-antiserum with the meal. **A)** Frequency of contractions in each time recorded along 240 mins after a meal. **B)** Comparison of the peristaltic wave rate of the crop along the first 90 mins after a meal. Each point represents Mean ± standard error. Asterisks represent statistically significant differences between treatments.

The amount of urine voided after feeding was significantly lower in 4^th^-instar larvae fed with anti-AT when compared with those insects fed with saline or with the preadsorbed AT antiserum (Fig 3A and 3B). The frequencies of contraction of the aorta and the crop peristaltic waves were significantly modified by ingestion of AT-antiserum (Figs 4 and 5). After a period of 90 mins during which the rate of waves was significantly decreased, crop contraction frequencies returned to control levels (Fig 4A and 4B). The behavior of the DV was similar, but covering a shorter period of time (Fig 5A and 5B).

### 3.5 Genomic characterization and expression of the *R. prolixus* AT receptor

**Fig 6:**
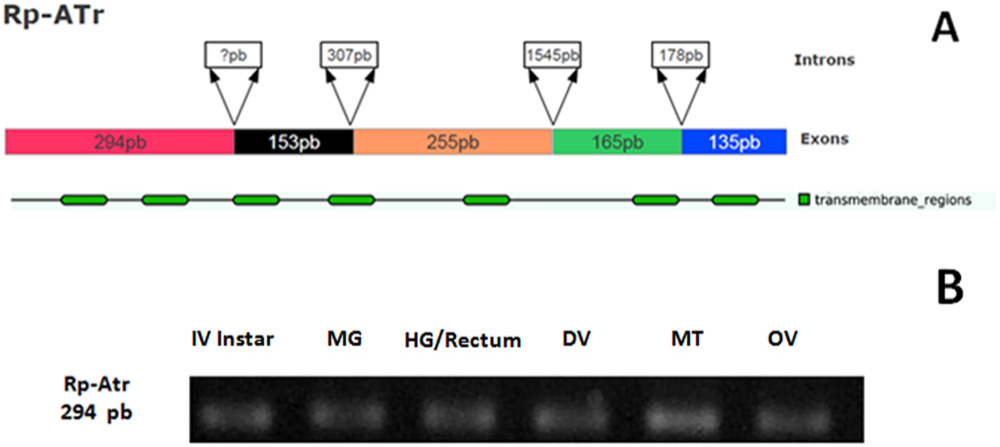
Characterization and expression of the *R*. *prolixus* AT receptor. **A)** Structure of the *RpATr* showing the existence of five exons and a predicted protein containing the typical seven transmembrane domains. **B)** *RpATr* expression in whole 4^th^-instar larvae and aorta, mid-gut and hindgut/rectum of adult.

The *R*. *prolixus* AT receptor is composed of five exons and four introns (Fig 6A). The predicted mature RNA encodes a 334 amino acid protein that includes the classical seven transmembrane domains characteristic of this family of receptors (Fig 6A; S1 File). The expression of the receptor was confirmed in several organs, including midgut, hindgut/rectum and DV (Fig 6B). Comparison of the fragment sequenced of the *R*. *prolixus* AT receptor with Allatotropin/orexin orthologous from other insect species revealed a high degree of conservation such as 60% of identity and 74% of similarity with *M*. *sexta* (S1 Fig).

## Discussion

*R*. *prolixus* and *T*. *infestans* are major vectors of Chagas disease in Latin America. Transmission occurs when insects feed, releasing together with urine and feces the infective form of *Trypanosoma cruzi*. Understanding the regulation of diuresis is therefore critical. The use of specific antiserums provided with the meal to alter the biology of blood-sucking vectors has been already demonstrated in several species [33–35], but not in triatominae insects. We have previously reported in the related species *T*. *infestans* that the injection of the AT-antiserum in the haemocoel, compared with insects that receive saline or a preadsorbed AT-antiserum solution, prevents the release of urine inhibiting the voiding of the rectum, showing that the presence of the antiserum in the haemocoel is able to specifically capture the peptide and block of its activity [24]. The experiments performed in this study show similar results strongly suggesting that, the antiserum administrated with the meal reaches the haemolymph, capturing the circulating peptide and inhibiting the ability of the rectum to release the urine, and also inhibiting the frequency of contractions of the DV. The possibility of delaying, or even blocking the elimination of urine and feces after a blood meal, provides new potential avenues for the control of diseases transmitted by Triatominae insects [24].

It has been previously reported the presence of allatotropic axons innervating the aorta, midgut and hindgut in *R*. *prolixus*. In these studies AT stimulated muscles in the ducts and the secretory portions of the salivary glands, increasing the expulsion of saliva, but failed to exert any myostimulatory effects on the digestive system or the DV under basal conditions [23]. In *T*. *infestans*, AT immunoreactivity is present in the aorta and the anterior midgut of 4^th^-instar larvae [27] and adults [25]. Allatotropic cells resembling open type secretory cells were found in the epithelial sheet of the crop in 4^th^-instar larvae and adults [25, 27]. In *T*. *infestans*, AT increased the contractions of the digestive tract (midgut and hindgut/rectum) and the DV [24, 25] only when the tissues were previously stimulated. AT regulatory activities on the peristaltic waves of the rectum were also confirmed by injecting 4^th^-instar larvae with anti-AT antiserum; a treatment that resulted in a decrease in the total quantity of urine eliminated [24]. In the present study we observed a similar phenomenon when 4^th^-instar larvae of *R*. *prolixus* were fed with a saline solution containing the same anti-AT antiserum; while controls fed with saline or AT-antiserum preadsorbed with the peptide displayed no differences in the quantity of urine voided. When the effect of feeding AT-antiserum was evaluated in adults, we observed a significant decrease in the peristaltic activity of the crop and the frequency of contractions of the aorta, showing that the antiserum was able to pass across the wall of the crop reaching the haemolymph. These results matched those of previous studies in *T*. *infestans*, that revealed myostimulatory activities of AT on the crop, associated with the presence of open type epithelial cells, which suggested that the peptide was acting in a paracrine mode [25].

The existence of daily rhythms in triatominae insects is widely documented [36, 37]. The basal frequency of aorta contractions displayed daily rhythmic contractions, with maximum frequencies in the afternoon when insects were preparing for blood-feeding. In *T*. *infestans*, the levels of AT in the midgut and MTs vary along a 24 h period, reaching their highest amounts a few hours previous to the beginning of the dark period when the insect is preparing for blood feeding [28].

AT did not display cardioacceleratory effects when it was assayed on non-stimulated tissues (basal frequency), neither when assayed on high nor low frequencies (i.e. morning and afternoon respectively), confirming results previously reported by Masood and Orchard [23]. However, we found that AT was very effective when applied after the DV has been previously stimulated with serotonin. These results confirm those previously described for *T*. *infestans*, reporting that AT synergizes the effect of serotonin [25]. This synergistic activity between serotonin and neuropeptides was also observed in *D*. *melanogaster* with the FMRFamide-related peptides. In fact, it was shown that the effect of FMRFamide varies when it is administered alone (having no effect), but causing an increment of the frequency of the aorta when it is applied together con serotonin [38].

The *R*. *prolixus* AT receptor is a GPCR that shares a high degree of sequence similarity with AT receptors from other insects [39–42]. A highly conserved sequence in the TM7 (YANSCAN[V/I/T]PI), which is considered a mammalian orexin receptor signature [36], is still recognisable in the insect receptors (S1 Fig). Although allatotropin and orexin are not structurally related, it is interesting that both peptides have been implicated with the regulation of food intake, starvation and energy metabolism, and their receptors seem to share a common ancestor [43]. The receptor, expressed in aorta, midgut and hindgut/rectum tissues, is the first AT receptor characterized for a hemimetabolous insect species, and supports the hypothesis that AT is acting as a myomodulator in *R*. *prolixus*, being involved in post-feeding diuresis. In summary all together in the present work we provide evidence that AT acting synergistically with serotonin plays a role as a myoregulatory and cardioacceleratory peptide in *R*. *prolixus*.

## Supporting Information

S1 Fig *RpATr* sequences from several holometabolous and hemimetabolous species. (PDF)

S1 File Comparison of *RpATr* genomic and cloned nucleotide sequences; and predicted amino acid sequence. Highlighted letters correspond to differences between predicted and cloned sequences.
(PDF)

S1 Movie Movie showing the anterograde contractions of the aorta (running from top to bottom in the movie) and the peristaltic waves of the crop, running in the opposite direction (from bottom to top in the movie). (MP4)
