# Supplementary figures and images for: Allatotropin Modulates Myostimulatory and Cardioacceleratory Activities in Rhodnius prolixus (Stal)
Source: PLoS One. 2015 Apr 21;10(4):e0124131. doi: 10.1371/journal.pone.0124131 (PMC4405368; doi:10.1371/journal.pone.0124131)

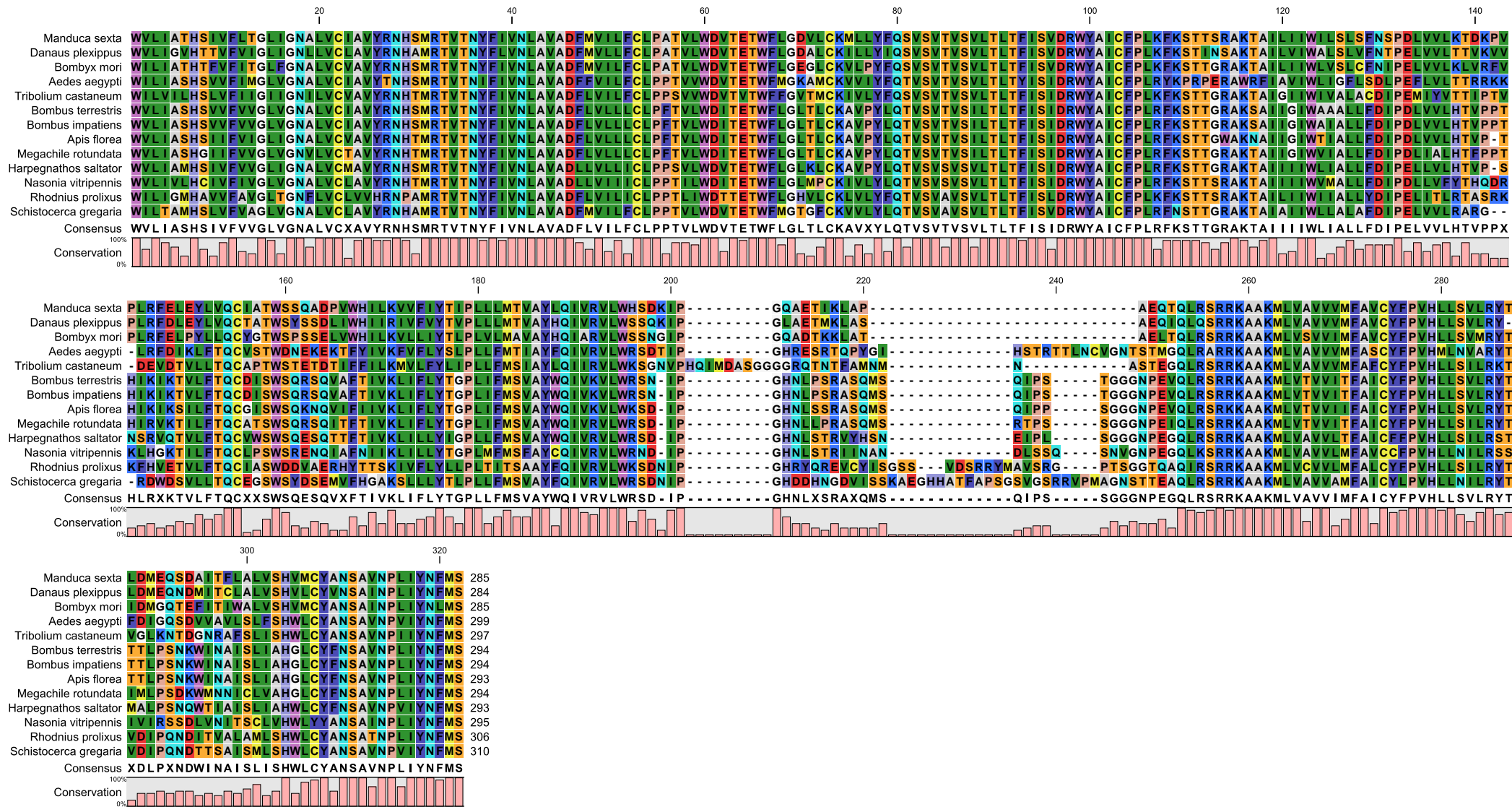

Supplement: S1 Fig — (PDF) [file pone.0124131.s001.pdf]
