# Supplementary material for: Allatotropin Modulates Myostimulatory and Cardioacceleratory Activities in Rhodnius prolixus (Stal)
Source: PLoS One. 2015 Apr 21;10(4):e0124131. doi: 10.1371/journal.pone.0124131 (PMC4405368; doi:10.1371/journal.pone.0124131)

>Rp_ATr protein sequence
MSDEDYLNMMTEHIFPTVYEWILIGMHAVVFAVGLTGNFLVCLVVHRNPAMRTVTNYFIVNLAVADFL
VILICLPPTLIWDTTETWFLGHVLCKLVLYFQTVSVAVSVLTLTFISLDRWYAICFPLKFKSTTSRAKTAILI
IWIIALLYDIPELITLRTASRKKFHVETVLFTQCIASWDDVAERHYTTSKIVFLYLLPLTITSAAYFQIVRVL
WKSDNIPGHRYQREVCYISGSSVDSRRYMAVSRGPTSGGTQAQIRSRRKAAKMLVCVVLMFALCYF
PVHLLSILRYTVDIPQNDITVALAMLSHWLCYANSATNPLIYNFMSGKSSITYP**stop**

>Rp_ATr genomic sequence
ATGTCCGATGAAGACTATCTGAACATGATGACCGAGCATATATTCCCTACGGTATACGAATGGATAT
TAATCGGCATGCACGCTGTAGTGTTTGCGGTCGGATTGACTGGCAACTTTCTAGTCTGTCTTGTTG
TTCATCGCAATCCGGCCATGAGAACCGTCACCAACTATTTCATCGTGAATCTGGCTGTAGCGGATT
TCTTAGTCATTTTGATATGTCTACCGCCAACATTGATATGGGATACCACAGAAACATGGTTTCTTGG
ACATGTCCTCTGCAAATTAGTGCTCTAC<__pb_>TTTCAGACAGTGTCCGTAGCGGTCAGCGTGC
TGACTCTTACATTCATATCATTAGATCGTTGGTATGCCATTTGCTTTCCATTAAAGTTCAAATCTACC
ACATCTCGAGCTAAAACTGCCATACTAATCATCTGGATAATCGCATTGTTGTAC<_307_pb_>GAT
ATTCCGGAGTTGATAACACTTCGTACAGCCAGTCGTAAGAAGTTTCACGTGGAAACGGTACTGTT
CACACAATGTATCGCCTCCTGGGACGATGTTGCCGAACGTCATTACACCACATCTAAAATTGTATT
TTTGTATTTACTGCCACTCACCATTACTTCTGCTGCTTACTTTCAAATTGTCAGAGTCCTTTGGAAG
TCGGACAACATACCTGGCCATCGCTATCAGCGAGAAGTTTGCTACATTTCAGGG<_1545_pb_>A
GCAGTGTAGATTCTAGAAGATACATGGCTGTATCCAGAGGACCTACATCCGGTGGCACACAGGCT
CAAATAAGATCTCGCAGGAAAGCTGCCAAAATGTTGGTGTGTGTGGTATTAATGTTCGCACTCTG
TTATTTTCCTGTACATCTGCTCAGTATTCTAAGA<_178_pb_>TATACTGTTGATATACCTCAAAAC
GACATCACTGTAGCCCTGGCAATGCTTTCACATTGGTTGTGTTATGCTAATTCGGCCACTAATCCT
CTTATCTACAATTTTATGTCAGGTAAAAGTTCTATAACGTATCCATGA

>Rp_ATr mRNA cloned
GATGACCGAGCATNTATTCCNTACNGTATACGAATGGATATTAATCGGCATGCACGCTGTAGTGTT
TGCGGTCGGATTGACTGGCAACTTTCTAGTCTGTCTTGTTGTTCATCGCAATCCGGCCATGAGAA
CCGTCACTAACTATTTCATCGTGAATCTGGCTGTAGCGGATTTCTTAGTCATTTTGATATGTCTACC
GCCAACATTGATATGGGATACCACAGAAACATGGTTTCTTGGACATGTCCTCTGCAAATTAGTGCT
CTACTTACAGACAGTGTCCGTAGCGGTCAGCGTGCTGACTCTTACATTCATATCATTAGATCGTTG
GTATGCCATTTGCTTTCCATTAAAGTTCAAATCTACCACATCTCGAGCTAAAACTGCCATACTAATC
ATCTGGATAATCGCATTGTTGTACGATATTCCGGAGTTGATAACACTTCGTACAGCCAGTCGTAAG
AAGTTTCACGTGGAAACGGTACTGTTCACACAATGTATCGCCTCCTGGGACGATGTTGCCGAACG
TCATTACACCACATCTAAAATTGTATTTTTGTATTTACTGCCACTCACCATTACTTCTGCTGCTTACT
TTCAAATTGTCAGAGTCCTTTGGAAGTCGGACAACATACCTGGCCATCGCTATCAGCGAGAAGTT
TGCTACATTTCAGGGAGCAGTGTAGATTCTAGAAGATACATGGCTGTATCCAGAGGACCTACATCC
GGTGGCACACAGGCTCAAATAAGATCTCGCAGGAAAGCTGCCAAAATGTTGGTGTGTGTGGTAT
TAATGTTCGCACTCTGTTATTTTCCTGTACATCTGCTCAGTATTCTAAGATATACTGTTGATATACCTC
AAAACGACATCACTGTAGCCCTGGCAATGCTTTCACATTGGTTGTGTTATGCTAATTCGNCNC

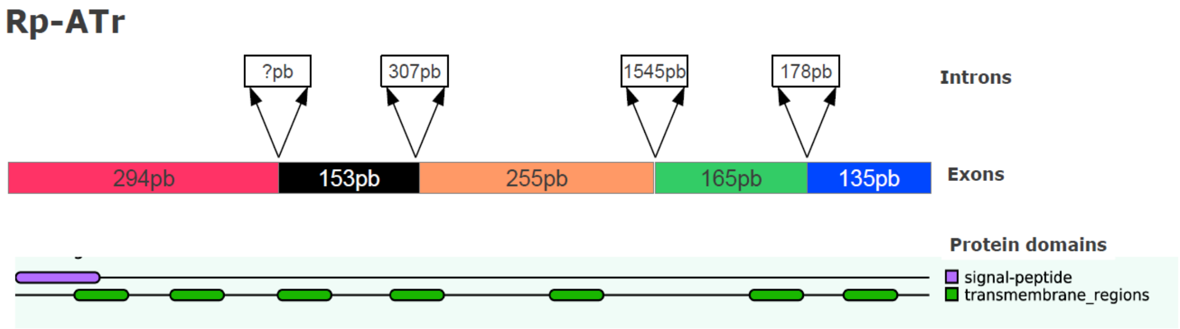

Supplement: S1 File — Highlighted letters correspond to differences between predicted and cloned sequences. (PDF) [file pone.0124131.s002.pdf]
